# Information literacy instruction for pharmacy students: a pharmacy librarian reflects on a year of teaching

**DOI:** 10.5195/jmla.2019.522

**Published:** 2019-01-01

**Authors:** Bridget C. Conlogue

**Affiliations:** Health Sciences Librarian for Pharmacy and Nursing, Nesbitt School of Pharmacy, Wilkes University, Wilkes-Barre, PA, bridget.conlogue@wilkes.edu

## Abstract

Librarians have ever-expanding teaching responsibilities in many academic disciplines. Assessment of learning outcomes requires longitudinal evaluation to measure true retention of skills and knowledge. This is especially important in the health sciences, including pharmacy, where librarians take an active role in teaching students to help prepare them for a profession in which solid information literacy skills are required to safely and effectively provide evidence-based care to patients. In this commentary, I reflect on a year of teaching in a pharmacy program and consider the outcomes of my instruction, areas for improvement, student retention of learning, assessment challenges, faculty-librarian collaboration, and continued support for library instruction in the pharmacy curriculum.

## INTRODUCTION

In May 2017, I began my position as health sciences librarian for pharmacy and nursing at Wilkes University. My position is embedded in the Nesbitt School of Pharmacy, and I report directly to the dean of pharmacy. I am the third librarian in this position and am fortunate to enjoy already established opportunities for teaching.

For a number of years, I have observed that students do not appear to retain skills and knowledge from library sessions over the long term. Through reading and reflecting, I have come to the conclusion that if I expect to develop and shape our students’ information literacy skills in a truly meaningful way, I must focus on enhanced classroom engagement and longitudinal learning and assessment. From the literature, I know librarians grapple continuously with these challenges and seek their resolution through a variety of teaching and assessment strategies. In this commentary, I reflect on these issues in the context of my experience teaching in a pharmacy program.

## LIBRARY INSTRUCTION

The pharmacy librarian is involved in teaching in two courses for first-year pharmacy students: “Foundations of Pharmacy Practice” and “Care Lab.” The library instruction session for the “Foundations of Pharmacy Practice” course placed much emphasis on formatting citations. The now retired librarian who developed and taught this session for many years commented in her notes that she was never satisfied with it and modified the session over the years, as did the second librarian in this position.

When I taught in this class in September 2017, I dispensed with the citation drill homework on the assumption that students could reference citation samples from the Pharmacy Research LibGuide. Instead, I tried a higher-learning-order approach and talked to the students about the connection between correctly formatted citations and academic honesty and scientific discovery. The session included reviewing the library website, the LibGuide, PubMed, and Proquest’s Health & Medical Collection, and creating an annotated bibliography for the major assignment in the course. The session was didactic and included a PowerPoint presentation and a web demonstration of resources. Part of a 1.5-hour class, this 45-minute session was designed to give students a broad overview of the library and its resources and to help orient them to important resources for their yearlong research project for the course. This was a lot of ground to cover in a short, one-off session.

I found that the students were not well engaged and did not respond readily to my questions. Although I did not conduct a formal assessment of the session, based on my observations, I believe its learning impact was low. When students approached me in late fall for assistance with their class projects, I asked if they recalled information from the library session. They did not. It was evident they were not referring to the Vancouver style page on the LibGuide that I had reviewed in class but instead were using Google to find sample citations. There might be students who did not ask for help, found appropriate references in the LibGuide, and, thus, did not need assistance; however, there was no way to know this without surveying the students. Therefore, I concluded that more work needed to be done on this session for fall 2018.

The next session I taught was in the “Care Lab” course in April 2018. “Care Lab” is a required course for first-, second-, and third-year students that focuses on teaching a wide variety of skills that students will need to practice pharmacy competently and safely. It is organized into weeklong segments taught by rotating faculty and instructors. As the “Care Lab” classroom seats twenty-four students, the first-year class of seventy-two students was broken into three sections; thus, I taught this session three times over the duration of a week. There were three third-year pharmacy student teaching assistants for the first-year course, with one for each section.

Historically, in the library instruction session, the librarian teaches on the topics of evidence-based medicine (EBM), information mastery, and PubMed. I wanted to keep a focus on these areas but decided to change the emphasis and delivery of the content. Previously, much of the PubMed instruction was provided before class via National Library of Medicine–created web tutorials, with some in-class review. Class time also involved setting up questions in population-intervention-comparator-outcome (PICO) format and reviewing disease-oriented versus patient-oriented evidence.

While these are important concepts to instill in future health care practitioners, it was, again, a lot of information to give to students in one session. Our students are bright and motivated, but all learners have limits, especially when there are so many things they are trying to learn at once. Also, from discussions with faculty, I knew that EBM and information mastery would be covered in other courses in subsequent years, so I felt comfortable scaling back on the scope of this content. To my knowledge, however, this would be the only time they would be taught how to search PubMed. Over the winter months, I worked with senior students on research projects and noticed that their skills in searching PubMed were minimal. I decided to refocus the lesson on PubMed, with less time spent structuring PICO questions.

“Care Lab” began at 8:00 a.m., and I observed that the students immediately paid attention and showed interest. Perhaps it was due to the smaller, more intimate classroom and the seating arrangement (six tables with four students at each, rather than theater-style seating). The cases and clinical questions used in the sessions were based on genuine scenarios and were vetted by the course director prior to the session, as I speculated that students would have more interest in real-world situations that involved actual practitioners and patients. When I spoke to students, I tried to impress upon them that knowing how to conduct effective searches will benefit them and their patients once they are in practice. Green and Ellis stated, “Adult learners need to know why they need to learn something before undertaking to learn it” [1]. Bruce et al. referred to this as the “Personal Relevance Frame” [2]. I continuously referred to the future, including references to them as “practitioners” in order to engage their “future-oriented” minds. Whether this was effective in shaping attitudes and behaviors remains to be seen.

The session began with a brief lecture with PowerPoint slides to explain the basic principles of EBM and article appraisal. After the thirty-five-minute lecture, the students, in pairs, completed a short exercise on paper in which they constructed a PICO-based, answerable clinical question from a simple case and answered some basic questions about the lecture content. After students completed the assignment, their papers were collected, and the teaching assistant led a review of the exercise, discussing each step briefly and asking students to call out their responses.

Next, the class performed a hands-on search in PubMed. I walked the students through each step of a simple search and explained Medical Subject Headings (MeSH) and other features. The students, still in pairs, were given another in-class assignment with a simple clinical question that required them to conduct their own searches in PubMed. They provided their search strategies and the citations and abstracts for three relevant articles. To encourage their information appraisal skills, they were asked to explain why they selected these articles. Assignment outcomes were mapped to the Association of College & Research Libraries Framework for Information Literacy [3] and School of Pharmacy educational outcomes.

During each session, the teaching assistant, as a peer of the students, added essential support to my commentary and offered her own experiences and thoughts. The teaching assistant and I walked around the room and answered students’ questions as they worked through both assignments. There was a good amount of discussion between the pairs and among the four students at each table. Completed PubMed search assignments were submitted before the end of the session through the course management system. I evaluated both assignments, for which scores were included in the final course grade. Unlike previous years, there was no assigned homework. One week later, students were asked to evaluate the session via an online survey.

The survey received an 89% response rate. Most (86%) students strongly agreed that “I learned at least one valuable skill in this lab,” 12% somewhat agreed, and 2% neither agreed nor disagreed. No students somewhat or strongly disagreed. The inclusion of active learning activities in the “Care Lab” session appeared to be a good strategy. Active learning is “[a]nything course-related that all students in a class session are called upon to do other than simply watching, listening and taking notes” [4]; fosters increased retention of content and improves practical application of skills in the clinical setting [5]; and simply makes classes more enjoyable and lively [4].

Indeed, teaching the “Care Lab” session was much more interesting than teaching the “Foundations” session due to the hands-on activities and increased interaction with the students. In the post-session online survey, students commented positively about the in-class activities: “Appreciated the hands-on activity at the end to apply the skills”; “[T]he exercises that we did helped me gain experience and practice using the databases”; and “I liked how she walked me through PubMed and had me do it on my own verus *[sic]* her just speaking to me.” Three students commented that they thought the session was unnecessary, as they believed themselves to already be proficient in online searching. Six students noted that they would have liked more hands-on activities (e.g., additional self-directed searches in PubMed). Also, three students suggested a printed handout with searching tips, which I found interesting as I assumed that students prefer digital format over print.

Although the survey results suggested an immediate increase in learning and overall positive response to the lab, I should not stop there and consider my class a success. Further, students receiving library instruction have varying levels of skills and exposure to previous library instruction and may be over-confident in self-reporting such skills [6, 7], which can make accurate learning outcomes difficult to determine. More work needs to be done to determine which skill was most valuable to them and to develop a plan to teach and evaluate searching skills as they progress into their second year and, importantly, into their third and fourth years when they are doing clinical rotations. If I conduct a PubMed searching session in “Care Lab” for first-year students and never follow up with additional sessions or reinforcement in subsequent years, it is unlikely and not very realistic to think that our students would have any real grasp of PubMed and other similar indexes. These are not designed for the casual user, and it takes many searches over time to use these resources effectively.

Studies show that despite our best efforts to introduce them to PubMed and proprietary indexes like Embase, students will very likely turn to public search engines (i.e., Google) [8, 9]. Practitioners, too, might use Google as a starting place for searching [10]. To address this, I briefly reviewed the caveats of using Google, Google Scholar, and other resources with students in the “Foundations” and “Care Lab” sessions. Additional instruction in this area may be appropriate and needed. There is much freely available high-quality medical and health information available on the web; thus, in addition to teaching our students how to conduct searches in secondary and tertiary resources, it is critical to teach them how to evaluate information that is freely available on the web.

An important part of our charge as librarians is to train students to evaluate and appraise what they find, wherever they find it. It seems futile to try to discourage students from using resources that they will probably use anyway. I explained to students in “Care Lab” that proficiency in PubMed and the ability to appraise and apply the primary literature to patient care is important. They will very likely be called upon by their preceptors to conduct research while on clinical rotations. Also, the pharmacist’s role in the provision of primary care is expanding, as is their active participation on the health care team in the clinical setting.

When students enter the workforce they may need to access and evaluate information beyond drug lookups. Their access to the proprietary, filtered, tertiary databases that they became familiar with and trained on while in school (e.g., DynaMed) might be limited or the databases might not be available to them in their future practice, depending on what their employer subscribes to or whether they are willing to pay. Additionally, I tell them they may not have access to a medical librarian for research assistance; again, this depends on their future employer.

## THE CHALLENGE: MAKING LIBRARY INSTRUCTION STICK

The ever-present challenge in library instruction, especially in health care education, is to develop and shape skills, knowledge, attitudes, and behaviors in our students from the time of instruction, throughout their long education, and beyond when they are working in real-world situations. There is concern about health care students’ ability to retain information-seeking skills and apply these later in the clinical setting [11, 12]. Carroll et al. stated that traditional one-shot information literacy lectures, such as my session for the “Foundations” class, foster a passive learning experience with low levels of skill retention, and they, therefore, integrated a flipped classroom model with pre-class and in-class active learning activities into their instruction [6]. The flipped classroom model is now very prevalent, and it was used by the former pharmacy librarians for teaching PubMed in the “Care Lab” course. In my previous work experience at a medical school, almost all library instruction was delivered via librarian-created online tutorials. Short-term student responses were positive, but there was little evidence of long-term retention, although no formal assessments were conducted.

How can we make library instruction “stick?” Pre- and post-class surveys measure immediate impact and are commonly conducted in library instruction. They are easily done when students are gathered together in a classroom setting. Former colleagues and I conducted several of these surveys, and I have seen them often in the literature and at poster sessions at conferences. How do we measure retention weeks, months, or a year after the session? Cmor et al. report disappointing results only two weeks post-instruction. They ask the important question of whether immediate improvement in knowledge and learning can be deemed successful if only a small percentage of students are able to apply what they learned at a later time. They assert that “performance-based” exercises and other standard assessments post-class, such as multiple-choice quizzes or self-assessments, do not evidence true (i.e. long-term) learning [8].

Longitudinal studies that measure retention are challenging to carry out because university students scatter after any given class [7]. However, as pharmacy curricula follow a cohort format, this could allow library instruction to be included in all four years, with opportunities to assess learning outcomes and retention in the same groups of students. Assessments could be conducted in the first year, again at the end of the second year, and so on, providing concrete data about skills and knowledge retention over time.

## CONCLUSION

Over the next year, I will continue to review and develop my instructional content. My first step will be to evaluate the responses provided in the “Care Lab” session evaluations and consider additional ways to improve this class. I may need to reintroduce the flipped classroom model. I also need to consider ways to improve the library session for “Foundations of Pharmacy Practice.”

In the future, I would like to incorporate some type of active learning into all of my library instruction, as evidence in the literature demonstrates its positive impact on learning outcomes. I will research assessments and teaching strategies used by other librarians and experiment with a blend of didactic, flipped classroom, and active learning. My goal is to continue to learn more about the pharmacy curriculum and to look for opportunities to develop library instruction across four years to support and enhance the topics that faculty teach.

I was invited to conduct a review of resources for a toxicology elective for fall 2018, and the course faculty and I discussed ways to enhance a simple didactic lecture to encourage better retention of the material. Designing meaningful assessments of learning will be a challenge, as I do not have a lot of experience in this area. In the fall, I joined the school’s assessment committee, which I expect to help develop and shape my skills. Our students are also trained in drug information seeking, which helps lay a base on which to develop strong searching skills and habits. Faculty buy-in is essential, and I am fortunate to have a lot of support here. Building the relationships to put all this into action takes time and effort, and I thank the founding pharmacy librarian for her work in this area. I believe I am off to a good start.
